# Tislelizumab plus concurrent chemoradiotherapy versus concurrent chemoradiotherapy for elderly patients with inoperable locally advanced esophageal squamous cell carcinoma: a multicenter, randomized, parallel-controlled, phase II clinical trial

**DOI:** 10.1186/s12885-025-13758-0

**Published:** 2025-02-25

**Authors:** Ke Zhang, Qifeng Wang, Jianzhong Cao, Chengcheng Fan, Wenbin Shen, Qin Xiao, Xiaolin Ge, Tian Zhang, Xiao Liu, Xi Chen, Jie Dong, Zewei Li, Zhunhao Zheng, Cihui Yan, Ping Wang, Qingsong Pang, Wencheng Zhang

**Affiliations:** 1 Department of Radiation Oncology, Tianjin Medical University Cancer Institute & Hospital, National Clinical Research Center for Cancer, Key Laboratory of Cancer Prevention and Therapy, Tianjin’s Clinical Research Center for Cancer, Huanhuxi Road, Hexi District, Tianjin, 300060 China; 2 Radiation Oncology Key Laboratory of Sichuan Province, Department of Radiation Oncology, Sichuan Cancer Hospital Institute, Sichuan Cancer Center, School of Medicine, University of Electronic Science and Technology of China, Chengdu, China; 3 Department of Radiotherapy, Shanxi Province Cancer Hospital/Shanxi Hospital Affiliated to Cancer Hospital, Chinese Academy of Medical Sciences/Cancer Hospital Affiliated to Shanxi Medical University, Taiyuan, 030013 China; 4 Department of radiation oncology, The Affiliated Cancer Hospital of Zhengzhou University & Henan Cancer Hospital, Zhengzhou, 450008 Henan China; 5 Radiotherapy Department of the Fourth Hospital of Hebei Medical University, Shijiazhuang, China; 6 Thoracic Radiotherapy Department Hunan Cancer Hospital the Affiliated Cancer Hospital of Xiangya School of Medicine, Central South University, Changsha, China; 7 Department of Radiation Oncology, Jiangsu Province Hospital and Nanjing Medical University First Affiliated Hospital, 300, Guangzhou Road, Nanjing, Jiangsu China; 8 Department of Nutrition Therapy, Tianjin Medical University Cancer Institute & Hospital, National Clinical Research Center for Cancer, Key Laboratory of Cancer Immunology and Biotherapy, Tianjin’s Clinical Research Center for Cancer, Huanhuxi Road, Hexi District, Tianjin, 300060 China; 9 Department of Immunology, Tianjin Medical University Cancer Institute & Hospital, National Clinical Research Center for Cancer, Key Laboratory of Cancer Immunology and Biotherapy, Tianjin’s Clinical Research Center for Cancer, Huanhuxi Road, Hexi District, Tianjin, 300060 China

**Keywords:** Concurrent chemoradiotherapy, Immunotherapy, Locally advanced esophageal squamous cell carcinoma, Older patients, Tislelizumab

## Abstract

**Background:**

The standard treatment for elderly patients with unresectable locally advanced esophageal squamous cell carcinoma (ESCC) is definitive chemoradiotherapy with S-1. However, the 3-year overall survival (OS) is limited to approximately 40%. Tislelizumab is the first- and second-line standard treatment for advanced ESCC with tolerable toxicity. In this study, we aimed to explore a new curative strategy for locally advanced unresectable ESCC in the elderly by combining tislelizumab with chemoradiotherapy.

**Methods:**

This study is an open-label, multicenter, investigator-initiated phase II clinical trial in older patients with inoperable locally advanced ESCC evaluating tislelizumab plus concurrent chemoradiotherapy compared with concurrent chemoradiotherapy. The main inclusion criteria were pathological confirmation of locally advanced inoperable ESCC at clinical cT1N2-3M0 or cT2-4bN0-3M0 (stage II–IVA), age ≥ 70 years, absence of previous systemic anti-tumor therapy, and adequate organ function. A total of 136 patients will be recruited from approximately seven centers (in Tianjin, Chengdu, Taiyuan, Zhengzhou, Shijiazhuang, Changsha, Nanjing) over a period of 18 months and randomized in a 1:1 ratio to receive tislelizumab in combination with concurrent chemoradiotherapy (tislelizumab + S-1 + radiotherapy) or concurrent chemoradiotherapy (S-1 + radiotherapy). The efficacy and safety of the treatment will be evaluated during the therapy and follow-up period until disease progression, death, or the end of the trial. The primary study endpoint was investigator-assessed progression-free survival (PFS), and secondary study endpoints were OS, objective response rate (ORR), duration of remission (DOR), and safety. Fresh or archival tumor tissues and peripheral blood samples will be used in exploratory studies.

**Discussion:**

This study is the first “programmed death-1 (PD-1) inhibitor combined with concurrent chemoradiotherapy” for elderly patients with inoperable locally advanced ESCC (NCT06061146). The synergistic efficacy of combined definitive concurrent chemoradiotherapy with tislelizumab is expected to result in survival benefits for elderly patients with inoperable locally advanced ESCC. Because S-1 plus concurrent radiotherapy is the standard treatment option for locally advanced ESCC in older patients, the combination of definitive concurrent chemoradiotherapy and tislelizumab has the potential to change the standard ESCC therapeutic strategy with comparable safety.

**Trial registration:**

ClinicalTrials.gov NCT06061146.Registered 9/10/2023.

**Supplementary Information:**

The online version contains supplementary material available at 10.1186/s12885-025-13758-0.

## Background

Esophageal cancer (EC) is a common malignant tumor with poor prognosis worldwide. In 2022, esophageal cancer became the seventh leading cause of cancer death globally, with 445,129 deaths [1]. The morbidity and mortality of esophageal cancer are 224,000 and 187,500 in China [2]. Esophageal squamous cell carcinoma (ESCC) is the primary histological subtype of EC [3]. The proportion of elderly patients with ESCC over the age of 70 years is as high as 30–40% [4], which is still increasing due to the aging trend of society. However, there are no consensus or guidelines and clinical trials for ESCC in the elderly.

To date, definitive radiotherapy is the main treatment for elderly patients with locally advanced ESCC because these patients are intolerable to surgery. However, the appropriate radiation field and dose remain controversial. Several recent studies showed that survival was not different between ESCC patients receiving involved field irradiation (IFI) and elective nodal irradiation (ENI) [5, 6]. In a retrospective analysis that enrolled 137 elderly ESCC patients aged 70 years or older, although there was no difference in the median overall survival (OS) and 3-year OS rates between the IFI and ENI groups, the incidence of grade ≥ 3 radiation esophagitis was lower in the IFI group [7]. In the exploration of radiation dose, 50.4 Gy is widely used in the concurrent chemoradiotherapy (CCRT) which is the standard care for locally advanced inoperable ESCC according to the results of RTOG94-05 study [8]. With the application of modern radiation techniques, several prospective randomized clinical studies revealed that a high dose (60 Gy) did not improve survival compared with 50 Gy, but increased grade ≥ 3 treatment-related adverse effects (TRAEs) [9–12]. Therefore, an IFI of 50 Gy in IFI might be preferred for elderly patients with ESCC.

Recent studies with small sample sizes demonstrated that the completion of dual-agent chemoradiotherapy (CRT) was limited in elderly patients with ESCC aged > 75 years because of the higher incidence of toxicities [13, 14]. S-1 is a new oral fluoropyrimidine derivative consisting of tegafur, 5-chloro-2,4-dihydroxypyridine, and potassium oxonate at a molar ratio of 1:0.4:1. S-1 may show a superior radiosensitizing effect compared with fluorouracil owing to its stable high-level plasma concentration [15]. 5-chloro-2,4-dihydroxypyridine inhibits DNA repair resulting from radiation-induced damage, enhancing the sensitivity of tumor cells to radiotherapy [16]. The results of multicenter phase II/III randomized controlled studies in elderly ESCC showed that combining S-1 and radiotherapy can not only improve survival but also tolerate toxicity compared with radiotherapy alone [16–18]. S-1 is administered orally, which is far more convenient than intravenous administration, particularly for elderly patients and those requiring nasal administration. Combining S-1 with radiotherapy has become the standard treatment strategy for elderly patients with locally advanced inoperable ESCC. However, survival was suboptimal, with a 3-year OS rate of approximately 40% [16–18]. New strategies are required to further improve treatment outcomes in elderly patients.

Emerging studies have indicated promising synergistic effects of combining immunotherapy with CRT, presenting a novel treatment avenue for ESCC [19–21]. The combination of radiotherapy and immunotherapy has the potential to enhance the anti-tumor efficacy. Radiotherapy can remodel the tumor microenvironment, and the addition of immunotherapy to radiotherapy likely synergizes with anti-tumor effects by accelerating tumor death, activating and maintaining anti-tumor immune capacity [22–24]. Furthermore, vascular normalization during combination therapy can ameliorate cellular hypoxia, thereby enhancing the lethality of radiation [22]. Tislelizumab, an anti-PD-1 antibody, has recently been proved in the first- and second-line standard treatment for advanced ESCC [25, 26]. The RATIONALE-302 study showed that tislelizumab had a good survival benefit in second-line treatment of advanced metastatic ESCC. Patients in the tislelizumab group showed prolonged median OS (8.6 months) and reduced TRAEs (73.3%) compared with patients in the chemotherapy group [26]. The RATIONALE-306 study also showed that tislelizumab in combination with chemotherapy achieved an ultralong survival benefit in the first-line treatment of advanced metastatic ESCC. The median OS and progression free survival (PFS) are 17.2 months and 7.3 months, respectively [25]. Tislelizumab exhibits mild toxicities [25, 26] and may be well tolerated in the elderly. However, the use of tislelizumab in combination with radiotherapy in elderly patients with ESCC remains unknown.

Herein, we conducted the first clinical trial to assess the efficacy and safety of tislelizumab plus concurrent S-1 combined with radiotherapy versus concurrent S-1 combined with radiotherapy in elderly patients with inoperable locally advanced ESCC. We recruited 136 patients with locally advanced esophageal squamous carcinoma within 18 months from seven centers: Tianjin Cancer Hospital, Sichuan Cancer Hospital, Fourth Hospital of Hebei Medical University, Henan Cancer Hospital, Shanxi Province Cancer Hospital, Jiangsu Province Hospital, and Hunan Cancer Hospital. There are more than 400 cases of radical radiotherapy for esophageal cancer at our center every year. The six sub-centers were tumor prevention and treatment centers in their regions. More than 600 patients with esophageal cancer undergo radical radiotherapy every year at Henan Cancer Hospital and Sichuan Cancer Hospital, and more than 200 patients with esophageal cancer undergo radical radiotherapy every year at Jiangsu Province Hospital. The total number of elderly esophageal cancer primary treatment patients treated at the seven centers exceeds 300 cases per year.

## Methods/design

### Study design

**Fig. 1:**
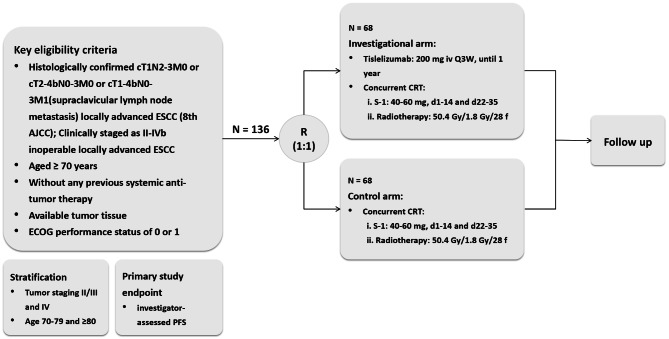
Study design. Elderly patients with locally advanced ESCC will be randomly divided into two groups: Tislelizumab combined with Concurrent CRT group (*N* = 68) and Concurrent CRT group (*N* = 68)

This trial is a multicenter, randomized, parallel-controlled, phase II study to assess the efficacy and safety of tislelizumab plus concurrent S-1 combined with radiotherapy versus concurrent S-1 combined with radiotherapy in elderly patients with inoperable locally advanced ESCC. The inclusion and exclusion criteria were as follows. Eligible patients will be randomized in a 1:1 ratio into the investigation and control groups. Randomization was stratified by age (70–79, ≥ 80 years) and clinical TNM staging (II, III, and IV). The CCRT is S-1 (40–60 mg, orally administered from day 1 to 14 and day 22 to 35) and radiotherapy (IFI) at 50.4 Gy/1.8 Gy/28 fractions (5 times per week). In the investigation group, in addition to the same CCRT regimen, tislelizumab was administered on the first day of radiotherapy at a dose of 200 mg intravenously for 30 min (not less than 20 min and not more than 60 min) per infusion and repeated every 21 days until disease progression or intolerance or up to a maximum of 1 year. The primary study endpoint was investigator-assessed PFS, and secondary study endpoints were OS, objective response rate (ORR), duration of remission (DOR), and safety. Figure 1 shows the diagram of the study design.

### Eligibility criteria for the trial

#### Inclusion criteria

1. Patients with histologically confirmed locally advanced ESCC staged as cT1N2-3M0, cT2-4bN0-3M0, or cT1-4bN0-3M1 (supraclavicular lymph node metastasis) according to the 8th edition of the American Joint Committee on Cancer; Clinically staged as II-IVb inoperable locally advanced ESCC (including contraindications to or refusal of surgery).
2. Patients aged ≥ 70 years, included male and female.
3. Patients with Eastern Cooperative Oncology Group Performance Status (ECOG PS) of 0 or 1.
4. Patients previously did not receive any systemic anti-tumor therapy, including systemic chemotherapy, radiotherapy, molecular targeted therapy, immunotherapy, biological therapy, topical therapy, and other investigational therapeutic agents.
5. Patients agreed to provide fresh or archival tumor tissues before and during concurrent chemoradiotherapy (after 40 Gy radiation) for biomarker analysis, such as programmed death ligand 1 (PD-L1). Fresh samples were used for this study. Tissue specimens were formalin-fixed paraffin-embedded (FFPE) for the exploration study.
6. Patients with adequate organ function, including renal, hepatic, and coagulation functions.
7. Patients with expected survival of ≥ 3 months.
8. Patients who have signed informed consent form.

#### Exclusion criteria

1. Patients have received surgery for ESCC.
2. Patients with esophageal fistulae because of invasion of the primary tumors.
3. Patients with risk of gastrointestinal bleeding, esophageal fistula or esophageal perforation.
4. Patients with poor nutritional status, weight loss ≥ 10% in the previous 2 months, and no significant improvement after nutritional intervention.
5. Patients with any active autoimmune disease or history of autoimmune disease (e.g., interstitial pneumonitis, uveitis, enteritis, hepatitis, hypophysitis, vasculitis, myocarditis, nephritis, hyperthyroidism, and hypothyroidism).
6. Patients had a history of organ/allogeneic transplantation, HIV infection, or other acquired or congenital immunodeficiency diseases.
7. Patients with active tuberculosis infection.
8. Patients with uncontrollable symptoms or diseases.
9. The investigator judged that the patients might not be suitable for treatment or could not complete the study.

### Planned sample size

The open-label, multicenter, parallel-controlled, investigator-initiated phase II clinical trial was expected to require 18 months to enroll all participants, with an additional follow-up period of 24 months after the last randomization. The median PFS was 16 months in elderly ESCC patients receiving concurrent S-1 combined with radiotherapy in previous studies [16–18]. Additionally, a phase II clinical study conducted at our center for locally advanced esophageal cancer with concurrent chemoradiotherapy versus concurrent chemoradiotherapy plus immunotherapy demonstrated that the 1-year and 2-year PFS rates for patients receiving concurrent chemoradiotherapy plus immunotherapy were 80.0% and 65.0%, respectively [19]. A single-arm phase II study of durvalumab and tremelimumab with definitive CCRT for locally advanced ESCC showed a 2-year PFS rate of 57.5% [21]. In addition, in a single-arm, multicenter, phase II trial of induction sintilimab and chemotherapy followed by concurrent chemoradiotherapy for locally advanced esophageal cancer, the 1- and 2-year PFS rates were 72.0% and 61.3%, respectively [27]. Therefore, we expected the median PFS to be 28 months at a hazard ratio of 0.57. The final sample size consisted of 136 patients by 1:1 randomized in the two groups with 80% power and one-sided alpha, and calculated for a 10% dropout rate. A prior multicenter phase III clinical trial of chemoradiotherapy for the treatment of ESCC in the elderly, in which our department participated, demonstrated a dropout rate of < 10%. Therefore, our assumption of a 10% dropout rate is justified. Contingency plans, such as extending the recruitment period, increasing study sites and recalculating the sample size, are implemented if the dropout rate exceeds the assumed 10% or if recruitment is not completed within the planned timeframe. Allocation to either arm was determined using a computer-generated randomization schedule. WZ generated a random allocation sequence and assigned all participants to the interventions.

### Study procedures

#### Evaluation and randomization

Patient participation in this study required informed consent before any study-specific screening or evaluation. As older people have impaired decisional capacity, legally authorized representatives can be involved in decision making. Screening examinations and assessments will be performed within 28 days prior to enrollment. Routine blood tests and blood biochemistry were performed prior to the start of the first cycle of treatment and were completed within 7 days prior to randomization. Patients must be restaged within four weeks prior to randomization, including undergoing computed tomography (CT) scans of the cervical, thoracic, and abdominal (including pelvic) CT or magnetic resonance imaging (all contrast-enhanced, replaced by routine CT scan if contrast is prohibited). Magnetic resonance imaging (MRI) of the brain is required when brain metastasis is suspected and diagnosed (replaced with contrast-enhanced CT if MRI is prohibited). Bone scan/Positron Emission Tomography (PET) is only performed when there are clinical indications with time to test relaxed within 42 days prior to the first dose of medication.

#### Intervention

In the treatment group, the patients received tislelizumab combined with concurrent S-1 and radiotherapy. Tislelizumab is administered via intravenous infusion every 3 weeks from the first day of radiotherapy until progressive disease or intolerance of concurrent chemoradiotherapy. Tislelizumab will be maintained for a maximum of one year. In the control group, the patients received concurrent S-1 and radiotherapy. TRAEs (such as vomiting, radiation esophagitis, leukopenia, checkpoint inhibitor pneumonitis, etc.) are treated symptomatically with antiemetic, analgesic, stimulation of granulocytopoiesis, and hormonal therapy, etc.

##### Chemotherapy

S-1 was orally administered from day 1 to 14 and day 22 to 35. The dose was based on the patient’s body surface area (BSA): 40 mg twice daily for BSA < 1.25 m^2^; 50 mg twice daily for 1.25 m^2^ ≤ BSA < 1.50 m^2^; and 60 mg twice daily for BSA ≥ 1.50 m^2^. If grade ≥ 3 hematologic, gastrointestinal, or dermal toxicity occurs during S-1 treatment, the dose need be decreased from 60, 50 to 40 mg, and S-1 will be discontinued immediately if a grade 4 TRAE occurs. In the event of hematological toxicity, such as leukopenia, neutropenia, or thrombocytopenia, the standard treatment involves symptomatic stimulation of leukocyte and platelet production therapies, close monitoring of patient’s blood routine, and implementation of infection prevention strategies.

##### Radiotherapy

Prior to patient enrollment, the radiation oncologist evaluated the patient’s thoracic CT or PET-CT to ensure that the treatment area did not significantly exceed the prescribed normal tissue limits and that the 50.4 Gy irradiation dose was appropriate for the patient. Radiotherapy is delivered using standardized intensity-modulated radiotherapy (IMRT) or volumetric rotational intensity-modulated radiotherapy (VMAT). The prescribed dose is total 50.4 Gy, with 1.8 Gy per fraction administered 5 times per week. IFI was used to determine the radiation field. Cone-beam CT verification is required once a week during treatment to ensure the accuracy of treatment. If a subject develops severe esophagitis or other serious radiation-related adverse events, comtinuation of radiotherapy needs to be evaluated by the investigator. Intensified supportive therapy is allowed to help the patient continue to receive radiotherapy without increased risk. For instance, in patients presenting with radiation esophagitis, we will undertake nasogastric tube placement, instruct the patient to refrain from ingesting food, and provide oral or intravenous pain management, should it be required.

##### Immunotherapy

In the investigational arm, tislelizumab was administered on the first day of radiotherapy at a dose of 200 mg intravenously for 30 min (not less than 20 min and not more than 60 min) per infusion and repeated every 21 days until disease progression or intolerance or up to a maximum of 1 year. Tislelizumab is infused during the day, either 3 days earlier or later. The ELDERS study, the first prospective cohort study of immunotherapy in older cancer patients, indicated that older cancer patients (70 + years of age) had no increased risk of high-grade toxicity compared with younger patients (< 70 years of age). Higher comorbidity burden and polypharmacy are associated with the occurrence of TRAEs [28]. In the event of immune-related adverse events (irAEs), including checkpoint inhibitor pneumonitis, hyperthyroidism, hypothyroidism, hypophysitis, enteritis, and myocarditis, occurring during the treatment period, the investigator is required to promptly provide the patient with appropriate symptomatic treatments, such as oral or intravenous hormone therapy. Treatment with tislelizumab is suspended when grade ≥ 3 irAEs occur.

#### Follow-up

Routine blood examination, biochemistry, and thyroid function were rechecked 30 days after the last dose. Survival status and tumor imaging will be evaluated every 3 months from the end of treatment for 2 years and every 6 months after 2 years. If the patient is too weak to return to the hospital on time, screening examinations can be performed at the local hospital as an alternative, but is not recommended. We will arrange for doctors to provide online medical consultations and remind elderly participants to return to the hospital on time for treatment and checkups in order to improve treatment outcomes and study quality. Transportation subsidies will also be provided to elderly patients to improve the completion of study protocols and reduce dropout rates. In addition, Comprehensive Geriatric Assessment (CGA) will be used for geriatric assessments during screening and follow-up. Notably, staff in all research centers will be required to undergo detailed training to ensure uniformity in treatment protocols, data collection, and reporting. Grade ≥ 3 TRAEs were reported to the main center and managed uniformly. We will also establish data-monitoring committees to monitor safety and protocol adherence.

### Outcome measures/endpoints

#### Primary endpoint

PFS is measured from the date of randomization until progression (as per RECIST 1.1 assessment, regardless of continued treatment) or death from any cause. Subjects who did not experience disease progression or death will be censored at the date of their last evaluable tumor assessment.

#### Secondary endpoints

OS is defined as the time from randomization to the date of death from any cause. For subjects who are alive, their survival time will be censored at the date of the last contact (“last known alive date”). OS was censored for subjects at the date of the last confirmed survival if they were lost to follow-up.

Objective response rate (ORR) is the proportion of randomized subjects who achieve the best response of complete response (CR) or partial response (PR) using the RECIST 1.1. Best objective response (BOR) is the best investigator-rated designation for the period between the date of enrollment and the date of objectively documented progression according to RECIST 1.1 criteria or to the initiation of subsequent anti-tumor therapy, whichever occurs first. For subjects with no progression and no initiation of subsequent anti-tumor therapy, the BOR will be determined based on all response evaluations.

Duration of Objective Response (DOR) is the time between the date of the first documented response (CR or PR) to the date of the first documented progression as determined by RECIST 1.1, or death due to any cause, whichever occurs first. For subjects who did not progress or die, the DOR was censored.

The severity of TRAEs was assessed using the Common Terminology Criteria for Adverse Events v5.0. All TRAEs were recorded from randomization to 90 days after the end of treatment, regardless of their relatedness to the study medication. For TRAEs, investigators should follow up until resolution, remission, or baseline levels, ≤ grade 1, steady state, or with a reasonable explanation (e.g., loss to follow-up, death). Chemoradiotherapy-related adverse effects such as esophagitis, pain, and nausea during CCRT can lead to decreased adherence in older patients, raising the likelihood of treatment interruption. The occurrence of grade ≥ 3 irAEs (e.g., checkpoint inhibitor pneumonitis, myocarditis, hypophysitis, etc.) can lead to the interruption or cessation of immunotherapy, which in turn affects treatment efficacy.

The investigators used a series of questionnaires to assess the quality of life of the elderly subjects before and after treatment in terms of multiple dimensions such as dietary conditions, nutritional status, mental status, social support, and comorbidity burden, etc. The questionnaires include the European Organization for Research and Treatment of Cancer Quality of Life Questionnaire (EORTC QLQ) C30&OES18, Charlson Comorbidity Index, Barthel Index, Instrumental Activities of Daily Living Scale, Mini-Nutritional Assessment, Mini-Mental State Examination, Geriatric Depression Scale, and Medical Outcomes Study Social Support Survey (Supplementary tables).

### Biomarker analysis and translational research

Serial biopsies will be collected from the primary site at three time points, if possible (before treatment, after 40 Gy of radiotherapy, and progression). Fresh or archived tumor tissue samples are recommended within 6 months (fresh samples preferred). The sample types were formalin-fixed, paraffin-embedded (FFPE) tumor tissue blocks or at least five FFPE tumor tissue sections of 4 μm thickness. Owing to factors such as TRAEs, elderly patients are less likely to undergo gastroscopic biopsy during treatment. Retention of fresh tumor tissue during treatment is case-specific and not mandatory. Paired pathology slides were analyzed using immunohistochemistry. In addition, peripheral blood was collected at three time points (before treatment, after 40 Gy of radiotherapy, and after 50 Gy of radiotherapy) to analyze the phenotype of immune-competent cells using flow cytometry.

### Statistical analysis

This study was an open-label, randomized, parallel-controlled phase II clinical study, subject to performance bias due to the absence of blinding. To mitigate selection bias, close subject contact was maintained, and the rate of lost visits and nonresponse was reduced. To mitigate detection bias, a uniform Case Report Form was used to collect data at each center. Furthermore, a computer program was used to generate the assignment list. Randomization was stratified according to age (70–79, ≥ 80 years) and clinical TNM staging (II, III, and IV) to reduce the incidence of confounding bias.

Time to event distributions (i.e., PFS and OS) were estimated using the Kaplan–Meier method, and log-rank tests were used to determine significance. Cox multivariable regression was used to determine the prognostic factors for survival benefit. Multiple factor analysis can be used for continuous variables to control for confounders. In terms of safety, we tabulated the incidence of each AE. Because drop-out and loss to follow-up are inevitable, collecting complete data records is difficult. Therefore, we constructed the following four analysis sets and performed efficacy and safety analyses. (A) Intention to treat: all subjects randomized to the study (whether or not they have received study drug) constitute this analysis set. (B) Full analyse set: subjects who received the study drug at least once after randomization according to the Intention-to-treat principle. (C) Per Protocol Set: a subset of the full analyse set. Subjects with protocol violations that were judged to have a significant impact on efficacy were excluded from this set. (D) Safety Set: all subjects who have received at least one treatment with the study drug (whether or not they participated in a randomized group) constitute the Safety Set for this study. χ2 test was used to determine whether the missingness was at random. When missingness was confirmed as at random, we will introduce the weighting function for weighting adjustment and calculate the weighted log-rank statistic. If there are many missing values, multiple complete data sets can be generated using the multiple inputation, weighted log-rank tests can be performed separately, and finally the results can be combined. Moreover, the R project will be applied for post hoc subgroup analysis and interaction tests. The relationship between different baseline characteristics (age, sex, ECOG PS, Charlson Comorbidity Index, nutritional status, dietary status, smoking or drinking status, tumor length, location, and lymph node status) and survival will be demonstrated in the form of forest plots. Subgroup analyses of previous phase III clinical trials have shown that age, sex, comorbidity burden, and nutritional status were associated with different outcomes with CCRT in terms of OS [16, 17]. Comorbidities and poor nutritional status could increase the incidence of TRAEs and reduce the completion of treatment in elderly patients, which in turn affects treatment efficacy.

## Discussion

This multicenter, randomized, parallel-controlled, phase II clinical trial aimed to enroll 136 elderly patients with inoperable locally advanced ESCC and to assess the efficacy and safety of tislelizumab plus concurrent S-1 and radiotherapy versus concurrent S-1 and radiotherapy. It is the first “PD-1 inhibitor combined with concurrent chemoradiotherapy” for elderly patients with inoperable locally advanced ESCC, with the aim of exploring a novel treatment strategy for elderly ESCC (NCT06061146). Notably, this study addresses the specific population of elderly ESCC patients, who will be assessed multidimensionally through a series of questionnaires before, during and after treatment. In addition, we will adopt a multidisciplinary collaboration (radiation oncology, medical oncology, nutrition, gerontology, and endoscopy departments, etc.) during the treatment period in order to provide individualized treatment to elderly patients. The trial was a randomized open-label clinical trial. Age, sex, smoking or drinking status and TNM staging are possible confounding variables that can be controlled for by randomization. In terms of limitations, unlike blinded trials, open-label trials are subject to ascertainment bias in the assessment of outcome measures because the investigators and participants were aware of the treatment assignment. Moreover, our study targeted elderly ESCC patients, which did not include elderly esophageal adenocarcinoma patients. However, the incidence of esophageal adenocarcinoma is higher in regions such as North America and Northern Europe [3], which may affect global promotability of the trial.

If tislelizumab plus concurrent S-1 and radiotherapy significantly improves the PFS of elderly patients with ESCC, further confirmatory phase III trials are planned. The results of our study may inform the optimization of treatments combining immunotherapy and concurrent chemoradiotherapy for elderly patients with inoperable locally advanced ESCC. Chemoradiotherapy combined with immunotherapy is expected to provide greater survival benefit for elderly ESCC patients, replacing CCRT as the new standard.

## Electronic supplementary material

Below is the link to the electronic supplementary material.

Supplementary Material 1

## Data Availability

The datasets during the current study can be obtained from the corresponding author upon reasonable request.

## Abbreviations

EC: Esophageal cancer
ESCC: Esophageal squamous cell carcinoma
IFI: Involved field irradiation
ENI: Elective nodal irradiation
CCRT: Concurrent chemoradiotherapy
TRAEs: Treatment-related advent events
irAEs: Immune-related adverse events
CRT: Chemoradiotherapy
PD-1: Programmed death-1
PFS: Progression-free survival
ECOG PS: Eastern Cooperative Oncology Group Performance Status
PD-L1: Programmed death ligand 1
FFPE: Formalin-fixed paraffin embedded
CT: Computed tomography
MRI: Magnetic resonance imaging
PET: Positron Emission Tomography
BSA: Body surface area
IMRT: Intensity-modulated radiotherapy
VMAT: Volumetric Modulated Arc Therapy
ORR: Objective response rate
CR: Complete response
PR: Partial response
BOR: Best objective response
DOR: duration of remission
EORTC QLQ: European Organization for Research and Treatment of Cancer Quality of Life Questionnaire
CGA: Comprehensive Geriatric Assessment
