# Supplementary material for: Tislelizumab plus concurrent chemoradiotherapy versus concurrent chemoradiotherapy for elderly patients with inoperable locally advanced esophageal squamous cell carcinoma: a multicenter, randomized, parallel-controlled, phase II clinical trial
Source: BMC Cancer. 2025 Feb 25;25:347. doi: 10.1186/s12885-025-13758-0 (PMC11863415; doi:10.1186/s12885-025-13758-0)
Supplement: Supplementary file 1 — Supplementary Material 1 [file 12885_2025_13758_MOESM1_ESM.pdf]

## Supplementary Table 1. Charlson comorbidity index (CCI) score

The CCI score was calculating using International Classification of Diseases, 9th Revision (ICD-9) codes.

| Comorbidity | Score |
|---|---|
| Prior myocardial infarction | 1 |
| Congestive heart failure | 1 |
| Peripheral vascular disease | 1 |
| Cerebrovascular disease | 1 |
| Dementia | 1 |
| Chronic pulmonary disease | 1 |
| Rheumatologic disease | 1 |
| Peptic ulcer disease | 1 |
| Mild liver disease | 1 |
| Diabetes | 1 |
| Cerebrovascular (hemiplegia) event | 2 |
| Moderate-to-severe renal disease | 2 |
| Diabetes with chronic complications | 2 |
| Cancer without metastases | 2 |
| Leukemia | 2 |
| Lymphoma | 2 |
| Moderate or severe liver disease | 3 |
| Metastatic solid tumor | 6 |
| Acquired immune-deficiency syndrome (AIDS) | 6 |

## Supplementary Table 2. EORTC QLQ-C30 (version 3)

We are interested in somethings about you and your health. Please answer all of the questions yourself by circling the number that best applies to you. There are no "right" or "wrong" answers. The information that you provide will remain strictly confidential.

Please fill in your initials:

Your birthdate (Day, Month, Year):

Today's date (Day, Month, Year):

---

| | Not at All | A Little | Quite a Bit | Very Much |
|---|---|---|---|---|
| 1. Do you have any trouble doing strenuous activities, like carrying a heavy shopping bag or a suitcase? | 1 | 2 | 3 | 4 |
| 2. Do you have any trouble taking along walk? | 1 | 2 | 3 | 4 |
| 3. Do you have any trouble taking a short walk outside of the house? | 1 | 2 | 3 | 4 |
| 4. Do you need to stay in bedora chair during the day? | 1 | 2 | 3 | 4 |
| 5. Do you need help with eating, dressing, washing yourself or using the toilet? | 1 | 2 | 3 | 4 |

### During the past week:

| | Not at All | A Little | Quite a Bit | Very Much |
|---|---|---|---|---|
| 6. Were you limited in doing either your work or other daily activities? | 1 | 2 | 3 | 4 |
| 7. Were you limited in pursuing your hobbies or other leisure time activities? | 1 | 2 | 3 | 4 |
| 8. Were you short of breath? | 1 | 2 | 3 | 4 |
| 9. Have you had pain? | 1 | 2 | 3 | 4 |
| 10. Did you need to rest? | 1 | 2 | 3 | 4 |
| 11. Have you had trouble sleeping? | 1 | 2 | 3 | 4 |
| 12. Have you felt weak? | 1 | 2 | 3 | 4 |
| 13. Have you lacked appetite? | 1 | 2 | 3 | 4 |
| 14. Have you felt nauseated? | 1 | 2 | 3 | 4 |
| 15. Have you vomited? | 1 | 2 | 3 | 4 |
| 16. Have you been constipated? | 1 | 2 | 3 | 4 |

**During the past week:**

| | Not at All | A Little | Quite a Bit | Very Much |
|---|---|---|---|---|
| 17. Have you had diarrhea? | 1 | 2 | 3 | 4 |
| 18. Were you tired? | 1 | 2 | 3 | 4 |
| 19. Did pain interfere with your daily activities? | 1 | 2 | 3 | 4 |
| 20. Have you had difficulty in concentrating on things, like reading a newspaper or watching television? | 1 | 2 | 3 | 4 |
| 21. Did you feel tense? | 1 | 2 | 3 | 4 |
| 22. Did you worry? | 1 | 2 | 3 | 4 |
| 23. Did you feel irritable? | 1 | 2 | 3 | 4 |
| 24. Did you feel depressed? | 1 | 2 | 3 | 4 |
| 25. Have you had difficulty remembering things? | 1 | 2 | 3 | 4 |
| 26. Has your physical condition or medical treatment interfered with your family life? | 1 | 2 | 3 | 4 |
| 27. Has your physical condition or medical treatment interfered with your social activities? | 1 | 2 | 3 | 4 |
| 28. Has your physical condition or medical treatment caused you financial difficulties? | 1 | 2 | 3 | 4 |

**For the following questions please circle the number between 1 and 7 that best applies to you**

29. How would you rate your overall health during the past week?

1 2 3 4 5 6 7

Very poor Excellent

30. How would you rate your overall quality of life during the past week?

1 2 3 4 5 6 7

Very poor Excellent

## EORTC QLQ - OES18

Patients sometimes report that they have the following symptoms or problems. Please indicate the extent to which you have experienced these symptoms or problems during the past week. Please answer by circling the number that best applies to you.

| During the past week: | Not at All | A Little | Quite a Bit | Very Much |
|---|---|---|---|---|
| 31. Could you eat solid food? | 1 | 2 | 3 | 4 |
| 32. Could you eat liquidised or soft food? | 1 | 2 | 3 | 4 |
| 33. Could you drink liquids? | 1 | 2 | 3 | 4 |
| 34. Have you had trouble with swallowing your saliva? | 1 | 2 | 3 | 4 |
| 35. Have you choked when swallowing? | 1 | 2 | 3 | 4 |
| 36. Have you had trouble enjoying your meals? | 1 | 2 | 3 | 4 |
| 37. Have you felt full up too quickly? | 1 | 2 | 3 | 4 |
| 38. Have you had trouble with eating? | 1 | 2 | 3 | 4 |
| 39. Have you had trouble with eating in front of other people? | 1 | 2 | 3 | 4 |
| 40. Have you had a dry mouth? | 1 | 2 | 3 | 4 |
| 41. Did food and drink taste different from usual? | 1 | 2 | 3 | 4 |
| 42. Have you had trouble with coughing? | 1 | 2 | 3 | 4 |
| 43. Have you had trouble with talking? | 1 | 2 | 3 | 4 |
| 44. Have you had acid indigestion or heartburn? | 1 | 2 | 3 | 4 |
| 45. Have you had trouble with acid or bile coming into your mouth? | 1 | 2 | 3 | 4 |
| 46. Have you had pain when you eat? | 1 | 2 | 3 | 4 |
| 47. Have you had pain in your chest? | 1 | 2 | 3 | 4 |
| 48. Have you had pain in your stomach? | 1 | 2 | 3 | 4 |

## Supplementary Table 3. Barthel Index of Activities of Daily Living

***Instructions:*** Choose the scoring point for the statement that most closely corresponds to the patient's current level of ability for each of the following 10 items. Record actual, not potential, functioning. Information can be obtained from the patient's self-report, from a separate party who is familiar with the patient's abilities (such as a relative), or from observation. Refer to the Guidelines section on the following page for detailed information on scoring and interpretation.

### The Barthel Index

Bowels
0 = incontinent (or needs to be given enemata)
1 = occasional accident (once/week)
2 = continent
*Patient's Score:* ____________________

Bladder
0 = incontinent, or catheterized and unable to manage
1 = occasional accident (max. once per 24 hours)
2 = continent (for over 7 days)
*Patient's Score:* ____________________

Grooming
0 = needs help with personal care
1 = independent face/hair/teeth/shaving (implements provided)
*Patient's Score:* ____________________

Toilet use
0 = dependent
1 = needs some help, but can do something alone
2 = independent (on and off, dressing, wiping)
*Patient's Score:* ____________________

Feeding
0 = unable
1 = needs help cutting, spreading butter, etc.
2 = independent (food provided within reach)
*Patient's Score:* ____________________

Transfer
0 = unable – no sitting balance
1 = major help (one or two people, physical), can sit
2 = minor help (verbal or physical)
3 = independent
*Patient's Score:* ____________________

Mobility
0 = immobile
1 = wheelchair independent, including corners, etc.
2 = walks with help of one person (verbal or physical)
3 = independent (but may use any aid, e.g., stick)
*Patient's Score:* ____________________

Dressing
0 = dependent
1 = needs help, but can do about half unaided
2 = independent (including buttons, zips, laces, etc.)
*Patient's Score:* ____________________

Stairs
0 = unable
1 = needs help (verbal, physical, carrying aid)
2 = independent up and down
*Patient's Score:* ____________________

Bathing
0 = dependent
1 = independent (or in shower)
*Patient's Score:* ____________________

**Supplementary Table 4. The Lawton Instrumental Activities of Daily Living Scale**

A. Ability to Use Telephone

1. Operates telephone on own initiative; looks up and dials numbers .................................................... 1
2. Dials a few well-known numbers............................ 1
3. Answers telephone, but does not dial .................... 1
4. Does not use telephone at all ..................................0

B. Shopping

1. Takes care of all shopping needs independently...1
2. Shops independently for small purchases ............ 0
3. Needs to be accompanied on any shopping trip.... 0
4. Completely unable to shop......................................0

C. Food Preparation

1. Plans, prepares, and serves adequate meals independently ................................................ 1
2. Prepares adequate meals if supplied with ingredients ........................................................0
3. Heats and serves prepared meals or prepares meals but does not maintain adequate diet ..................... 0
4. Needs to have meals prepared and served ...........0

D. Housekeeping

1. Maintains house alone with occasion assistance (heavy work).............................................................. 1
2. Performs light daily tasks such as dishwashing, bed making.............................................................. 1
3. Performs light daily tasks, but cannot maintain acceptable level of cleanliness............................... 1
4. Needs help with all home maintenance tasks ...... 1
5. Does not participate in any housekeeping tasks .. 0

E. Laundry

1. Does personal laundry completely ......................... 1
2. Launders small items, rinses socks, stockings, etc .. 1
3. All laundry must be done by others ........................ 0

F. Mode of Transportation

1. Travels independently on public transportation or drives own car ....................................................... 1
2. Arranges own travel via taxi, but does not otherwise use public transportation ........................1
3. Travels on public transportation when assisted or accompanied by another .................................... 1
4. Travel limited to taxi or automobile with assistance of another ...............................................0
5. Does not travel at all................................................ 0

G. Responsibility for Own Medications

1. Is responsible for taking medication in correct dosages at correct time........................................... 1
2. Takes responsibility if medication is prepared in advance in separate dosages.............................0
3. Is not capable of dispensing own medication ....... 0

H. Ability to Handle Finances

1. Manages financial matters independently (budgets, writes checks, pays rent and bills, goes to bank); collects and keeps track of income ........................ 1
2. Manages day-to-day purchases, but needs help with banking, major purchases, etc ........................ 1
3. Incapable of handling money ................................ 0

---

Scoring: For each category, circle the item description that most closely resembles the client's highest functional level (either 0 or 1).

## Supplementary Table 5. Mini Nutritional Assessment

Complete the screen by filling in the boxes with the appropriate numbers. Total the numbers for the final screening score.

### Screening

**A Has food intake declined over the past 3months due to loss of appetite, digestive problems, chewing or swallowing difficulties?**
0=severe decrease in food intake
1=moderate decrease in food intake
2=no decrease in food intake

**B Weight loss during the last 3 months**
0=weight loss greater than 3kg (6.6lbs)
1=does not know
2=weight loss between 1 and 3kg(2.2 and 6.6 lbs)
3=no weight loss

**C Mobility**
0=bed or chair bound
1=able to get out ofbed /chair but does not go out
2=goes out

**D Has suffered psychological stress or acute disease in the past 3months?**
0=yes 2=no

**E Neuropsychological problems**
0=severe dementia or depression
1=mild dementia
2=no psychological problems

**F Body Mass Index(BMI)=weightin kg/(height in m)²**
0=BMI less than 19
1=BMI 19 to less than 21
2=BMI 21 to less than23
3=BMI 23 or greater

**Screening score (subtotal max.14 points)**
12-14 points: Normal nutritional status
8-11 points: At risk of malnutrition
0-7 points: Malnourished
For a more in-depth assessment,continue with questions G-R

**G Lives independently(not in nursing home or hospital)**
1=yes 0=no

**H Takes more than 3 prescription drugs per day**
0=yes 1=no

**I Pressure sores or skin ulcers**
0=yes 1=no

**J How many full meals does the patient eat daily?**
0=1 meal
1=2 meals
2=3meals

**K Selectedconsumption markers for protein intake**
At least oneserving of dairy product (milk,cheese,yoghurt) per day yes/no
Two or more servings or of legumes or eggs per week yes/no
Meat, fish or poultry every day yes/no
0.0=if 0 or 1 yes
0.5=if 2 yes
1.0=if 3 yes

**L Consumes two or more servings of fruit or vegetables per day?**
0=no 1=yes

**M How much fluid (water,juice,coffee,tea,milk...) is consumed per day?**
0.0=less than 3 cups
0.5=3 to 5 cups
1.0=more than 5 cups

**N Mode of feeding**
0=unable to eat withoutassistance
1=self-fed with some difficulty
2=self-fed without any problem

**O Self view of nutritional status**
0=views self as being malnourished 1=is uncertain of nutritional state
2=views self as having no nutritional problem

**P In comparison with other people of the same age, how does the patient consider his/her health status?**
0.0=not as good
0.5=does not know
1.0=as good
2.0=better

**Q Mid-arm circumference(MAC)in cm**
0.0=MAC less than21
0.5=MAC21 to 22
1.0=MAC greater than 22

**R Calf circumference (CC) in cm**
0=CC less than 31
1=CC 31 or greater

**Assessment (max.16 points)**
**Screening score**
**Total Assessment (max.30 points)**

**Malnutrition Indicator Score**
24 to 30 points Normal nutritional status
17 to 23.5 points At risk of malnutrition
Less than 17 points Malnourished

**Supplementary Table 6.** **Mini-Mental State Examination (MMSE)**

Patient's Name:______________________________ Date:______________

**Instructions: Ask the questions in the order listed.**
**Score one point for each correct response within each question or activity.**

| Maximum Score | Patient's Score | Questions |
|---|---|---|
| 5 | | "What is the year? Season? Date? Day of the week? Month?" |
| 5 | | "Where are we now: State? County? Town/city? Hospital? Floor?" |
| 3 | | The examiner names three unrelated objects clearly and slowly, then asks the patient to name all three of them. The patient's response is used for scoring. The examiner repeats them until patient learns all of them, if possible. Number of trials: ____________ |
| 5 | | "I would like you to count backward from 100 by sevens." (93, 86, 79,72, 65, ...) Stop after five answers. Alternative:"Spell WORLD backwards." (D-L-R-O-W) |
| 3 | | "Earlier Itold you the names of three things. Can you tell me what those were?" |
| 2 | | Show the patient two simple objects, such as a wristwatch and a pencil, and ask the patient to name them. |
| 1 | | "Repeat the phrase:'No ifs, ands, or buts.'" |
| 3 | | "Take the paper in your right hand, fold it in half, and put it on the floor." (The examiner gives the patient a piece of blank paper.) |
| 1 | | "Please read this and do what it says." (Written instruction is "Close your eyes.") |
| 1 | | "Make up and write a sentence about anything." (This sentence must contain a noun and a verb.) |
| 1 | | "Please copy this picture." (The examiner gives the patient a blank piece of paper and asks him/her to draw the symbol below. All 10 angles must be present and two must intersect.) 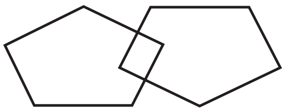 |
| 30 | | **TOTAL** |

## Interpretation of the MMSE

| Method | Score | Interpretation |
|---|---|---|
| Single Cutoff | <24 | Abnormal |
| Range | <2<br>1<br>>2<br>5 | Increased odds of dementia<br>Decreased odds of<br>dementia |
| Education | 21<br><2<br>3<br><2<br>4 | Abnormal for 8th grade education<br>Abnormal for highschool education<br>Abnormal for college education |
| Severity | 24-30<br>18-23<br>0-17 | No cognitive impairment<br>Mild cognitive impairment<br>Severe cognitive<br>impairment |

**Supplementary Table 7.** **Geriatric Depression Scale (short form)**

Choose the best answer for how you have felt over the past week:

1 . Are you basically satisfied with your life? YES / **NO**

2 . Do you often get bored? **YES** / NO

3. Do you feel that your situation is hopeless? **YES**/ NO

4. Do you feel pretty worthless the way you are now? **YES** / NO

5 . Do you prefer to stay at home, rather than going out and doing new things? **YES** / NO

Answers in bold indicate depression. Score 1 point for each bolded answer.

**Total Score :**____________

**Supplementary Table 8. Medical Outcomes Study Social Support Survey (Chinese Version)**

1. How many familiar family members and friends do you have?

   Please write down the number of familiar family members and friends: ____

**People sometimes look to others for companionship, assistance, or other types of support. How often is each of the following kinds of support available to you if you need it? Choose one number from each line.**

| | None of the time | A little of the time | Some of the time | Most of the time | All of the time |
|---|---|---|---|---|---|
| 2. Someone to help you if you were confined to bed | 1 | 2 | 3 | 4 | 5 |
| 3. Someone you can count on to listen to you when you need to talk | 1 | 2 | 3 | 4 | 5 |
| 4. Someone to give you good advice about a crisis | 1 | 2 | 3 | 4 | 5 |
| 5. Someone to take you to the doctor if you needed it | 1 | 2 | 3 | 4 | 5 |
| 6. Someone who shows you love and affection | 1 | 2 | 3 | 4 | 5 |
| 7. Someone to have a goodtime with | 1 | 2 | 3 | 4 | 5 |
| 8. Someone to give you information to help you understand a situation | 1 | 2 | 3 | 4 | 5 |
| 9. Someone to confide in or talk to about yourself or your problems | 1 | 2 | 3 | 4 | 5 |
| 10. Someone who hugs you | 1 | 2 | 3 | 4 | 5 |
| 11. Someone to get together with for relaxation | 1 | 2 | 3 | 4 | 5 |
| 12. Someone to prepare your meals if you were unable to do it yourself | 1 | 2 | 3 | 4 | 5 |
| 13. Someone whose advice you really want | 1 | 2 | 3 | 4 | 5 |
| 14. Someone to do things with to help you get your mind off things | 1 | 2 | 3 | 4 | 5 |
| 15. Someone to help with daily chores if you were sick | 1 | 2 | 3 | 4 | 5 |
| 16. Someone to share your most private worries and fears with | 1 | 2 | 3 | 4 | 5 |
| 17. Someone to turn to for suggestions about how to deal with a personal problem | 1 | 2 | 3 | 4 | 5 |
| 18. Someone to do something enjoyable with | 1 | 2 | 3 | 4 | 5 |

| | | | | | |
|---|---|---|---|---|---|
| 19. Someone who understands your problems | 1 | 2 | 3 | 4 | 5 |
| 20. Someone to love and make you feel wanted | 1 | 2 | 3 | 4 | 5 |
